# Disease characteristics and outcomes in patients with chronic kidney disease and type 2 diabetes: a matched cohort study of spironolactone users and non-users

**DOI:** 10.1186/s12882-020-01719-7

**Published:** 2020-02-26

**Authors:** Michael Blankenburg, Csaba P. Kovesdy, Anne-Kathrin Fett, Raymond G. Griner, Alain Gay

**Affiliations:** 1 Market Access, Public Affairs & Sustainability, Pharmaceuticals, Bayer AG, Berlin, Germany; 2 Division of Nephrology, University of Tennessee Health Science Center, Memphis, TN USA; 3 IQVIA Commercial GmbH & Co. OHG, Frankfurt, Germany; 4 IQVIA, Basel, Switzerland; 5 Medical Affairs & Pharmacovigilance, Pharmaceuticals, Bayer AG, Berlin, Germany

**Keywords:** Spironolactone, Mineralocorticoid receptor antagonist, Diabetic kidney disease, Real-world evidence, End-stage renal disease

## Abstract

**Background:**

Limited evidence has indicated that addition of a steroidal mineralocorticoid receptor antagonist (MRA) to the standard of care reduces proteinuria in patients with diabetic kidney disease (DKD); however, there are limited data regarding real-world MRA use in these patients. This study aimed to describe the characteristics of spironolactone users and non-users with DKD, and to explore their clinical outcomes.

**Methods:**

This was a non-interventional, retrospective cohort study using demographic and clinical data from a US claims database (PharMetrics Plus) and the Experian consumer data asset during 2006–2015. Baseline characteristics (e.g. comorbidities) and post-inclusion clinical outcomes were described in matched cohorts of spironolactone users and non-users (*n* = 5465 per group).

**Results:**

Although matching aligned key demographic and clinical characteristics of the cohorts, a significantly greater proportion of spironolactone users than non-users had oedema, proteinuria, and cardiovascular disease at baseline (*P* < 0.0001). During the post-inclusion period, disease progression and clinical events of interest such as acute kidney injury were more commonly observed in spironolactone users than non-users. Users also had higher healthcare resource utilization and costs than non-users; however, these differences diminished at later stages of disease.

**Conclusions:**

In this study, spironolactone users had a greater comorbidity burden at baseline than matched non-users, suggesting that the presence of certain comorbidities may be contributing factors in the decision to prescribe spironolactone. High healthcare resource utilization and costs for patients at later stages of disease, irrespective of spironolactone use, highlight the need for new therapies for DKD.

## Background

Diabetic kidney disease (DKD) is defined by the Kidney Disease Outcomes Quality Initiative and American Diabetes Association guidelines as a clinical diagnosis based on the presence of albuminuria (≥30 mg/g creatinine) and/or a reduced estimated glomerular filtration rate (< 60 mL/min/1.73 m^2^) in a patient with diabetes in the absence of other primary causes of kidney damage [1, 2]. It has previously been reported that 38.3% of people with type 2 diabetes (T2D) develop DKD, and 31.6% have evidence of kidney damage before or at the time of their T2D diagnosis [3, 4]. Although the proportion of patients with DKD among those with diabetes remains stable, the prevalence of DKD is increasing globally, driven primarily by the rising prevalence of T2D [5].

People with DKD have an increased risk of cardiovascular disease and death compared with those with T2D alone, including a two- to threefold-higher risk of fatal or non-fatal myocardial infarction [6–10]. Furthermore, those with DKD who progress to end-stage renal disease (ESRD) have a high mortality of 15–20% per year [11]. Treatment with an angiotensin-converting enzyme inhibitor (ACEi) or an angiotensin II receptor blocker (ARB) is the standard of care for prevention of disease progression in patients with DKD [1]. However, despite receiving treatment with an ACEi or ARB in addition to glucose- and lipid-lowering agents, individuals with DKD remain at high risk for cardiovascular events and progression to ESRD [12–15].

Overactivation of the mineralocorticoid receptor (MR) occurs under pathological conditions and contributes to hypertrophy, inflammation, and fibrosis, leading to cardiovascular and renal damage [16, 17]. Steroidal MR antagonists (MRAs) that inhibit this pathway, such as spironolactone or eplerenone, are recommended for the treatment of resistant hypertension and heart failure, both of which are common comorbidities of DKD [18–20]. Furthermore, results from a few small clinical trials have suggested that addition of an MRA to the standard of care in patients with mild-to-moderate chronic kidney disease (CKD) with or without diabetes may further reduce proteinuria; however, there is also an increased risk of hyperkalaemia. It is not known whether MRA therapy reduces the risk of ESRD or cardiovascular events in these patients [21–23].

There are limited data on the use of MRAs in routine clinical practice. In a previous observational study, we identified that real-world MRA use was low (1.2%) in patients with CKD; however, use increased with greater disease burden to 1.8% in those with DKD and 6.6% in those with DKD and heart failure. Almost all patients who received an MRA were prescribed spironolactone [24].

In the present study, we aimed to describe the characteristics of patients with DKD who received spironolactone compared with patients with DKD who did not receive spironolactone, and to explore clinical outcomes during the post-inclusion period.

## Methods

### Study design and data sources

This was a non-interventional, retrospective cohort study conducted using anonymized demographic and clinical data from the PharMetrics Plus (PMTX+) US claims database between January 2006 and December 2015. The aggregated PMTX+ database comprises adjudicated claims for more than 150 million unique patients across the United States (~ 40 million active in 2011) with both pharmacy and medical coverage. Additional data on imputed race/ethnicity and income were obtained for a subset of patients from the Experian consumer data asset using anonymous patient identifiers. Experian is a national marketing database including demographic, lifestyle, and financial attributes for approximately 300 million individuals.

### Matched cohorts of spironolactone users and non-users

Patients with diagnoses of both CKD and T2D (i.e. DKD) were identified in PMTX+ using International Classification of Diseases, Ninth and Tenth Revision, Clinical Modification (ICD-9-CM and ICD-10-CM) codes (Table S1).

**Fig. 1:**
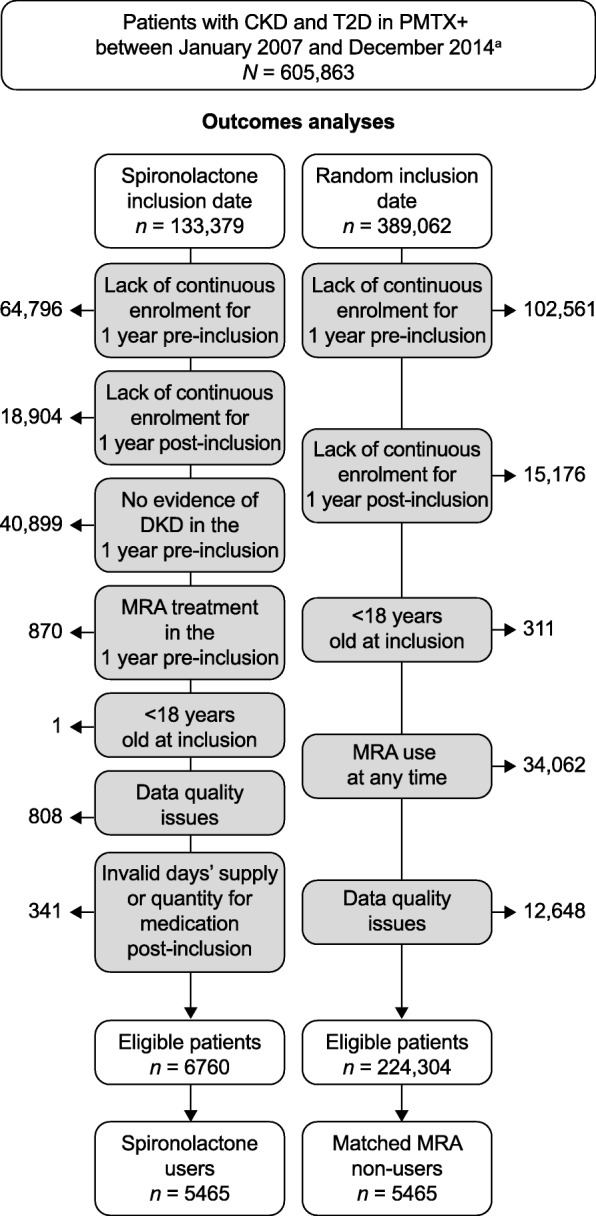
Summary of the matched cohorts of spironolactone users and non-users. ^a^Although the total study period was from January 2006 to December 2015, the inclusion window is smaller owing to the minimum data requirements pre- and post-inclusion date. CKD, chronic kidney disease; MRA, mineralocorticoid receptor antagonist; PMTX+, PharMetrics Plus; T2D, type 2 diabetes

Cohorts of spironolactone users and matched non-users were created (Fig. 1). The inclusion date for users was defined as the first claim for spironolactone between January 2007 and December 2014. Users were not permitted to have made a previous claim for an MRA for at least 1 year pre-inclusion. Non-users were assigned an inclusion date at a similar time in their disease progression to spironolactone users. Because users had their inclusion date on or after the first DKD claim, a random inclusion date within the inclusion window was selected for non-users as a proxy for the time post-diagnosis (Fig. S1A). This random date was required to be at least 1 year before the patient’s latest enrolment date and 1 year after their earliest enrolment date in the database. Non-users were not permitted to have received an MRA at any time.

To be eligible for either of the matched cohorts, patients had to have at least 1 year of data available pre- and post-inclusion date. Patients were excluded if they were younger than 18 years at the inclusion date or had data-quality issues associated with the health plan enrolment file that prohibited analysis (e.g. invalid enrolment dates or incomplete claims data).

From the eligible patients, spironolactone users and non-users were then matched based on the following parameters: age in years at inclusion (18–24, 25–34, 35–44, 45–54, 55–64, 65–75, ≥75), sex, CKD stage at inclusion, heart failure in 1 year pre-inclusion, hypertension in 1 year pre-inclusion, days since initial stage-specific diagnosis of DKD (≤90, 91–180, > 180) number of unique medications in 1 year pre-inclusion (< 10, ≥10), imputed race/ethnicity, income (≤$50,000, $50,001–75,000, >$75,000) and calendar quarter and year at inclusion.

### Variables

Baseline characteristics were extracted from the databases at inclusion, where available, or in the 1-year pre-inclusion period. Demographic variables extracted from PMTX+ included age, sex, and geographic region; imputed race/ethnicity and income were also obtained for those patients with available data in Experian. Clinical variables including CKD stage, Charlson comorbidity index (CCI) score, and comorbidities (e.g. cardiovascular disease, hyperkalaemia) were identified using ICD-9-CM, ICD-10-CM, and procedure codes.

Patients were followed within the post-inclusion period until the end of continuous health plan enrolment. Clinical outcomes were ascertained for the post-inclusion period of at least 1 year. Clinical events of interest included new heart failure events, acute kidney injury, diabetic retinopathy, stroke (any), hyperkalaemia, acute coronary syndrome, peripheral artery disease, proteinuria, stroke (ischaemic), hyponatraemia, reproductive system and breast disorders, revascularization, and amputation. Clinical events were identified using ICD-9-CM and ICD-10-CM codes. We aimed to evaluate incident rather than prevalent clinical events; therefore, to be included in the analysis as two events, a gap of 60 days was required between diagnoses of acute clinical events, and a gap of 360 days was required between diagnoses of chronic clinical events. Progression to a more advanced stage of CKD, to ESRD, or to renal replacement therapy (RRT) was identified by a diagnostic code (ICD-9-CM, ICD-10-CM, procedure codes) for any stage of CKD that was more advanced than the inclusion CKD stage.

Healthcare resource utilization and costs were ascertained for the post-inclusion period, and costs were also calculated at baseline using 1-year pre-inclusion data. Healthcare resource utilization consisted of inpatient, outpatient, and emergency department visits, with inpatient visits further subdivided into cardiovascular-related and DKD-related visits based on the primary diagnosis. Healthcare costs were calculated from the sum of the allowed amount on all claims. The allowed amount is the amount the health plan allows for a particular service and includes the paid amount plus any member liability. Total healthcare costs were subdivided into pharmacy, inpatient, and outpatient costs.

### Statistical analysis

Descriptive statistics were reported for all variables. Statistical comparisons between the matched groups were performed for baseline characteristics to ensure appropriate matching and were evaluated using McNemar (or McNemar–Bowker) tests for categorical variables and the Wilcoxon signed-rank test for continuous variables.

Outcome variables were described in each cohort using summary statistics for categorical and quantitative (continuous) data. Continuous data were described by median, mean, minimum, maximum, and interquartile range (IQR). Kaplan–Meier curves were created for time to disease progression. CKD progression, healthcare resource utilization, and costs in the post-inclusion period were reported for the overall matched cohorts and stratified by CKD stage at inclusion.

### Exploratory analysis

An exploratory analysis was conducted to better understand differences in baseline characteristics and outcomes based on spironolactone treatment persistence. A non-mutually exclusive cohort of patients with CKD and T2D who received spironolactone was generated. Patients were required to have at least 2 years of pre-inclusion data to ensure there was sufficient time since the initial stage-specific diagnosis of DKD. The inclusion date was defined as the first claim for spironolactone between January 2008 and December 2014 (Fig. S1B). The exclusion criteria for this cohort were the same as for the matched cohorts. The cohort was stratified by treatment persistence; non-persistent users included those who discontinued treatment within 6 months of initiation, whereas persistent users were treated for at least 6 months. Persistence was calculated based on time (in consecutive days) from the inclusion date until the first occurrence of discontinuation, medication switch, or the end of the respective follow-up period. Complete discontinuation was defined as a gap in the prescription of spironolactone of at least 60 days following the expected date of dispensation. A switch from spironolactone to eplerenone was defined as a claim for eplerenone within 60 days after the last day of supply of spironolactone; if a claim for eplerenone occurred at least 60 days after the last day of supply, then this constituted a switch following a treatment gap. Spironolactone therapy restart was defined as a refill of spironolactone after the minimum 60-day gap, with no evidence of eplerenone use. Baseline variables and outcomes were ascertained for this cohort as previously described for the matched cohorts.

## Results

### Baseline characteristics of matched cohorts

The matched cohorts of spironolactone users and non-users (*n* = 5465 per group) did not differ significantly with respect to demographics at inclusion; however, some differences remained with respect to clinical characteristics. A significantly greater proportion of users than non-users had cardiovascular disease (65.9% versus 62.1%), oedema (43.7% versus 25.2%), and proteinuria (22.7% versus 13.5%), while a significantly lower proportion of users than non-users had hyperkalaemia (10.2% versus 12.3%) (Table 1). Furthermore, 89.0% of users had a CCI score of 4 or higher, compared with 78.2% of non-users. Pre-inclusion median annual healthcare costs per person were significantly higher in users than in non-users ($10,436 versus $0).

**Table 1:** Baseline demographic and clinical characteristics of matched cohorts of spironolactone users and non-users

Characteristic	Spironolactone users (*n* = 5465)	Non-users (*n* = 5465)	*P* value^b^
Age at inclusion (years)			
Median (range)	62 (20–82)	62 (23–82)	0.0005
Sex (%)			
Male	60.5	60.5	#
Ethnicity^a^ (%)			
Caucasian	90.0	90.0	#
African American	6.7	6.7	#
Hispanic	2.6	2.6	#
Other	0.1	0.1	#
Unspecified	0.6	0.6	#
CKD stage at inclusion (%)			
Stage 1	3.4	3.4	#
Stage 2	9.1	9.1	#
Stage 3	38.8	38.8	#
Stage 4	6.8	6.8	#
Stage 5	0.4	0.4	#
ESRD/RRT	11.5	11.5	#
Missing	30.0	30.0	#
Comorbidities (%)			
Heart failure	48.6	48.6	#
Hypertension	98.6	98.6	#
CV disease	65.9	62.1	< 0.0001
Oedema	43.7	25.2	< 0.0001
Proteinuria	22.7	13.5	< 0.0001
Hyperkalaemia	10.2	12.3	0.0007
Annual pre-inclusion median healthcare costs (US$)			
Total costs	33,684	25,776	< 0.0001
Inpatient costs	10,436	0	< 0.0001
Outpatient costs	9398	8502	< 0.0001
Pharmacy costs	5721	5695	0.19
Medications of interest (%)			
ARBs	40.0	33.2	< 0.0001
ACEis	55.5	52.3	< 0.001

^a^Among the subset of patients linkable to the Experian database (*n* = 698 per group)

^b^*P* values calculated using McNemar (or McNemar–Bowker) tests for categorical variables and the Wilcoxon signed-rank test for continuous variables. Cases where perfect agreement exists between spironolactone users and non-users, owing to being included in the matching criteria, are identified by #

*ARB* angiotensin II receptor blocker, *ACEi* angiotensin-converting enzyme inhibitor, *CKD* chronic kidney disease, *CV* cardiovascular, *ESRD* end-stage renal disease, *RRT* renal replacement therapy

### Clinical events and disease progression in the post-inclusion period

**Fig. 2:**
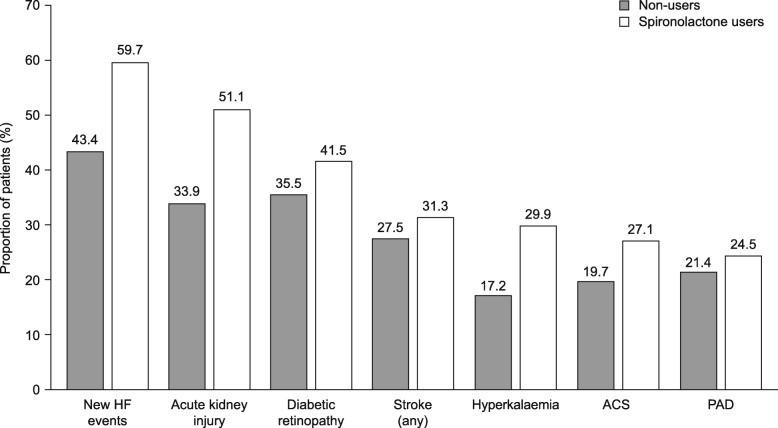
Clinical events of interest in the post-inclusion period in matched spironolactone users and non-users. A 60-day gap was used to count acute events (ACS, acute kidney injury, stroke [any], HF, and hyperkalaemia), and a 360-day gap was used to count chronic events (PAD and diabetic retinopathy). ACS, acute coronary syndrome; HF, heart failure; PAD, peripheral artery disease

**Fig. 3:**
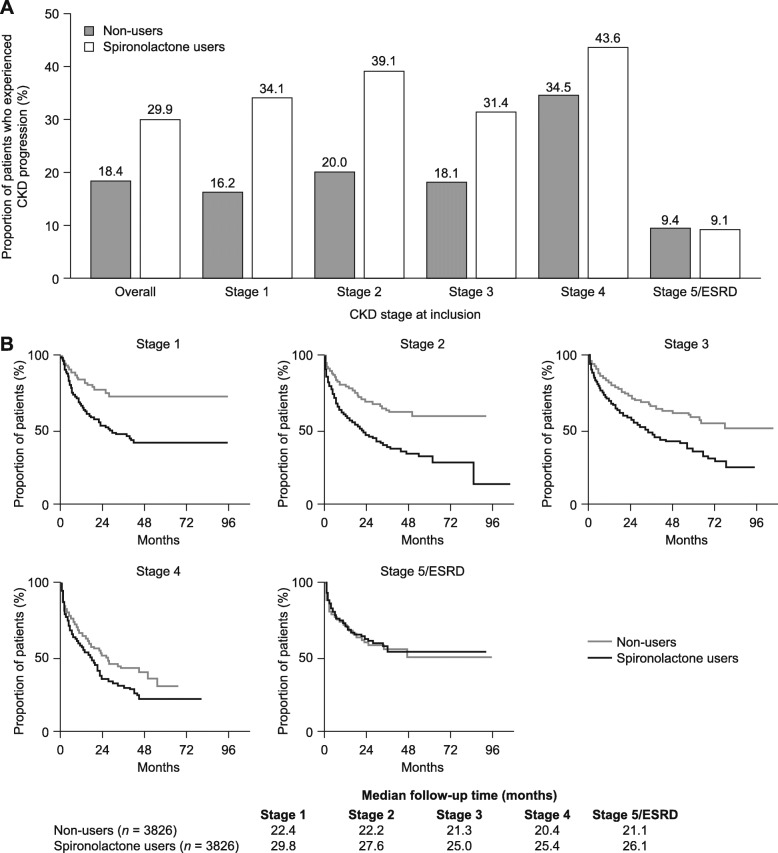
CKD progression in matched spironolactone users and non-users stratified by CKD stage at inclusion. (**A**) Proportion of patients who experienced progression to a more advanced stage of kidney disease (higher CKD stage, ESRD or renal replacement therapy) by 1 year post-inclusion. (**B**) Kaplan–Meier plots showing CKD progression in the matched cohorts during the post-inclusion period. CKD, chronic kidney disease; ESRD, end-stage renal disease

The median post-inclusion period was 786 (interquartile range [IQR] 549–1174) days for users and 641 (IQR 471–953) days for non-users. During the post-inclusion period, 39.2% and 53.9% of spironolactone users and 33.1% and 49.3% of non-users received ARBs and ACEis, respectively. A larger proportion of users than non-users experienced clinical events of interest (Fig. 2 and Fig. S2), including acute kidney injury (51.1% versus 33.9%) and hyperkalaemia (29.9% versus 17.2%). After 1 year post-inclusion, the proportion of users and non-users who had progressed to a more advanced stage of kidney disease (higher stage, ESRD, or RRT) was 29.9% and 18.4%, respectively. When stratified by CKD stage at inclusion, the difference in disease progression between the cohorts was less pronounced at advanced stages (Fig. 3).

### Healthcare resource utilization and costs in the post-inclusion period

**Fig. 4:**
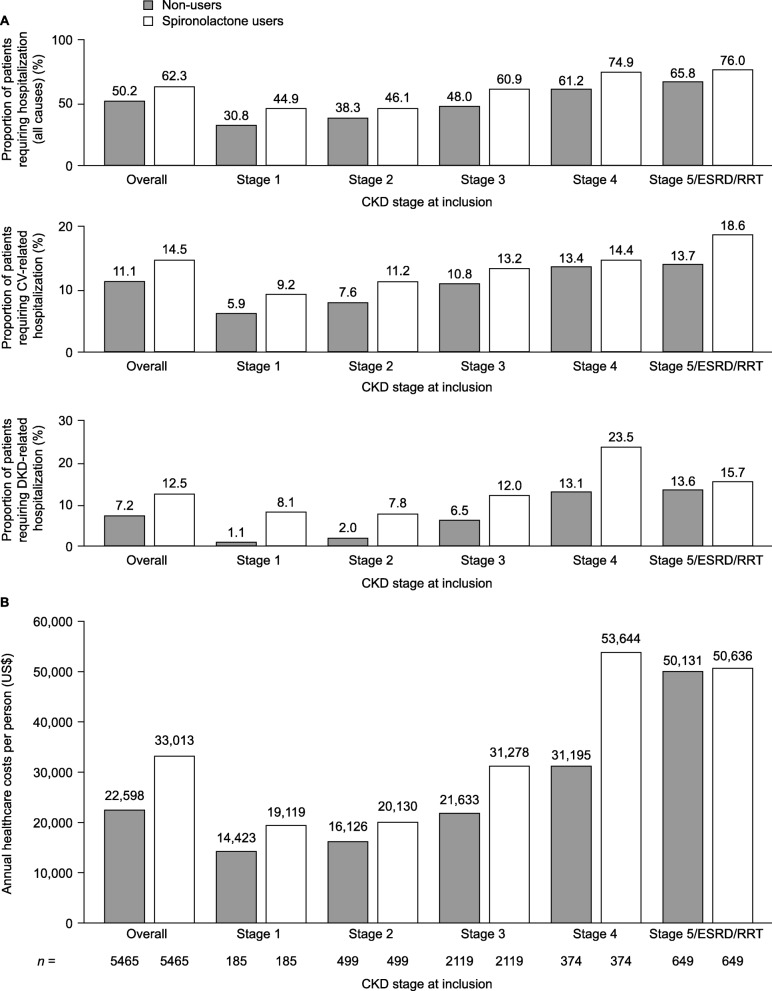
Healthcare resource utilization and costs in matched spironolactone users and non-users. (**A**) Proportion of patients hospitalized in the post-inclusion period stratified by CKD stage at inclusion. (**B**) Total median post-inclusion healthcare costs stratified by CKD stage at inclusion. CKD, chronic kidney disease; CV, cardiovascular; DKD, diabetic kidney disease; ESRD, end-stage renal disease; RRT, renal replacement therapy

Almost all users (99.7%) and non-users (99.6%) had at least one post-inclusion outpatient visit, whereas a greater proportion of users (64.2%) than non-users (55.1%) visited the emergency department. Users were more commonly hospitalized during the post-inclusion period than non-users, including all-cause, cardiovascular-, and DKD-related hospitalizations (Fig. 4A). Greater proportions of users than non-users were hospitalized at all CKD stages, although for all-cause and DKD-related hospitalizations, the differences between the cohorts were smaller for patients at CKD stage 5/ESRD/RRT at inclusion than for the overall cohorts.

Overall, annual median total healthcare costs per person in the post-inclusion period were highest for users than for non-users (Fig. 4B). Total healthcare costs were higher for users at CKD stage 1–4 at inclusion, with the largest difference between the cohorts observed at CKD stage 4. Healthcare costs were similar between users and non-users at CKD stage 5/ESRD/RRT.

### Spironolactone treatment persistence

An exploratory analysis investigated the baseline characteristics and clinical outcomes of spironolactone users (*n* = 5430) stratified by treatment persistence into persistent and non-persistent users. A number of differences were observed in the baseline demographic and clinical characteristics of the cohorts, including a greater comorbidity burden for non-persistent users than for persistent users and higher pre-inclusion median healthcare costs (Table S2). Progression to a more advanced stage of kidney disease (higher stage, ESRD, or RRT) by 1 year post-inclusion occurred in 23.1% of persistent users and 31.7% of non-persistent users (Fig. S3). In the post-inclusion period, non-persistent users more commonly experienced clinical events of interest than persistent users (Fig. S4). Annual median healthcare costs remained higher for non-persistent users than for persistent users ($36,879 versus $26,837) in the post-inclusion period.

## Discussion

There are limited data regarding the real-world use of MRAs, particularly in patients with DKD. This study builds upon the findings of a previous retrospective study investigating MRA use in patients with CKD with or without diabetes and/or heart failure, and focuses specifically on the comparison of baseline characteristics and outcomes between patients with DKD who are users or non-users of spironolactone [24]. After matching for key demographic and clinical characteristics, including the approved indications for spironolactone use (heart failure and hypertension), we found that spironolactone users were more severely ill than non-users at baseline. This is indicated by the higher proportions of users than non-users with oedema, proteinuria, and cardiovascular disease, which were not included in the matching criteria, and by the greater proportion of users than non-users with a CCI score of 4 or higher. Therefore, our findings suggest that spironolactone users have a greater comorbidity burden than non-users. Some of the differences between the cohorts at baseline may reflect the variables that were taken into consideration by physicians when deciding whether to prescribe MRAs. For example, the lower proportion of users than non-users with hyperkalaemia at baseline could be explained by a reluctance to prescribe spironolactone to patients with a history of hyperkalaemia, as hyperkalaemia is a known side effect of spironolactone treatment [21].

During the post-inclusion period, we observed that a larger proportion of users than non-users experienced clinical events of interest and CKD progression. Correspondingly, users had higher healthcare resource utilization and costs than non-users. However, the observed differences in outcomes between the cohorts are difficult to interpret with confidence, because the clinical differences observed at baseline may result in confounding. These results are also in contrast to the findings of a real-world study by Yang et al., which identified a lower risk of progression to ESRD in spironolactone users than non-users [25]. This may be explained by differences in the study populations, as the Yang et al. study included patients with CKD stage 3/4 with or without diabetes, and their population was less severely ill than the population included in the present study. In both studies, hyperkalaemia occurred more commonly in users than in non-users during the post-inclusion period.

Interestingly, the differences in outcomes between users and non-users diminished for patients at advanced stages of CKD. For example, for patients at CKD stage 5/ESRD at inclusion, progression to ESRD or RRT was reported for 9.1% of users and 9.4% of non-users. A similar trend was observed for all-cause hospitalization, DKD-related hospitalization, and total healthcare costs. For these outcomes, smaller differences were observed between users and non-users at CKD stage 5/ESRD/RRT at inclusion than with the overall cohorts. A potential explanation for this trend is that, at later stages of disease, spironolactone is likely to only be prescribed to patients who are tolerant of MRA therapy; therefore, these patients are less likely to experience adverse drug reactions and more likely to experience benefits from spironolactone treatment. In general, healthcare use and costs were high irrespective of spironolactone use or non-use for patients at CKD stage 5/ESRD/RRT; for example, annual costs were approximately US$50,000 per patient in both cohorts.

The exploratory analysis revealed better clinical outcomes in persistent than in non-persistent users in the post-inclusion period, including fewer clinical events and a lower proportion experiencing disease progression. This may be explained by differences in patient characteristics, as those who persisted with spironolactone treatment were less severely ill at baseline than those who discontinued within 6 months. The reasons for treatment discontinuation in this cohort are unknown but may be related to the incidence of adverse drug reactions such as hyperkalaemia [26]. Alternatively, it could be hypothesized that more severely ill patients may experience fewer beneficial effects and therefore terminate treatment earlier. However, causality cannot be inferred from these data.

The main strength of this longitudinal study is the use of data from a large cohort of patients with DKD who are representative of the US commercially insured population in terms of age and sex. However, there are several limitations that need to be considered. First, these results are not generalizable to the global DKD population because elderly individuals are under-represented in PMTX+, and there are no available data on non-US based patients. Secondly, this is a descriptive study without adjustment for clinical characteristics, such as the presence of oedema, proteinuria, and cardiovascular disease. The difference between the proportion of users and non-users with cardiovascular disease at baseline (65.9% versus 62.1%) will bias interpretation of outcomes occurring during the post-inclusion period owing to residual confounding, but is not expected to influence the results unduly. Lastly, as this is an observational study, causality cannot be inferred between spironolactone treatment and outcomes.

Further limitations arise from the use of claims data, without access to complete medical records. The diagnoses of CKD and T2D cannot be confirmed; therefore, these cohorts can be considered only a proxy for a DKD population. Moreover, diagnoses will have included both incident and prevalent cases. Exposure to spironolactone was inferred from prescriptions, with no information available regarding adherence to the prescribed regimen. It should also be noted that some outcomes may be under-recorded in claims databases; particularly the results of laboratory tests, which may result in under-estimation of proteinuria and inaccuracies in determination of CKD and CKD stage [27].

## Conclusions

These data suggest that patients with DKD who are prescribed spironolactone have a greater comorbidity burden than those who do not receive an MRA. Patients at advanced stages of disease (CKD stage 5/ESRD/RRT) have a high medical and economic burden irrespective of spironolactone use; this observation is particularly important given the increasing prevalence of DKD [5] and highlights the need for new therapies.

## Supplementary information

**Additional file 1 Table S1.** List of codes used to identify eligible patients with CKD and T2D. **Table S2.** Baseline demographic and clinical characteristics of the cohort of all patients receiving spironolactone, overall and stratified by spironolactone treatment persistence. **Figure S1.** Determination of inclusion date for study cohorts. (A) Matched users and non-users of spironolactone with DKD. (B) Cohort of spironolactone users for exploratory analysis of treatment persistence. CKD, chronic kidney disease; DKD, diabetic kidney disease; T2D, type 2 diabetes. **Figure S2.** Other clinical events of interest occurring in the post-inclusion period in the matched cohorts of spironolactone users and non-users. A 60-day gap was used to count acute events (stroke [ischaemic], revascularization, and hyponatraemia), a 360-day gap was used to count chronic events (proteinuria and reproductive system and breast disorders), and a 1-day gap was used for other events (amputation). **Figure S3.** Progression to a more advanced stage of CKD, ESRD, or RRT in 1-year post-inclusion in persistent and non-persistent users of spironolactone. CKD, chronic kidney disease; ESRD, end-stage renal disease; RRT, renal replacement therapy. **Figure S4.** Clinical events of interest occurring in the post-inclusion period in persistent and non-persistent users of spironolactone. A 60-day gap was used to count acute events [ACS, acute kidney injury, stroke (any), HF and hyperkalaemia] and a 360-day gap was used to count chronic events (PAD and diabetic retinopathy). ACS, acute coronary syndrome; AKI, acute kidney injury; HF, heart failure; PAD, peripheral artery disease.

## Data Availability

The datasets used were obtained from the IQVIA Real-World Data Adjudicated Claims database, hereafter referred to as PharMetrics Plus (IQVIA, Durham, North Carolina, USA). This is a closed database for which the authors had administrative permission to use. The datasets analysed during the current study are available from the corresponding author on reasonable request and with permission of IQVIA.

## Abbreviations

ACEi: Angiotensin-converting enzyme inhibitor
ARB: Angiotensin II receptor blocker
CCI: Charlson comorbidity index
CKD: Chronic kidney disease
DKD: Diabetic kidney disease
ESRD: End-stage renal disease
IQR: Interquartile range
MRA: Mineralocorticoid receptor antagonist
PMTX+: PharMetrics Plus
RRT: Renal replacement therapy
T2D: Type 2 diabetes
