# Supplementary material for: Disease characteristics and outcomes in patients with chronic kidney disease and type 2 diabetes: a matched cohort study of spironolactone users and non-users
Source: BMC Nephrol. 2020 Feb 26;21:61. doi: 10.1186/s12882-020-01719-7 (PMC7045439; doi:10.1186/s12882-020-01719-7)

**Table S1** List of codes used to identify eligible patients with CKD and T2D

| ICD code | Description |
| --- | --- |
| Type 2 diabetes | |
| 250.00 | Diabetes mellitus without mention of complication, type II or unspecified type, not stated as uncontrolled |
| 250.02 | Diabetes mellitus without mention of complication, type II or unspecified type, uncontrolled |
| 250.10 | Diabetes with ketoacidosis, type II or unspecified type, not stated as uncontrolled |
| 250.12 | Diabetes with ketoacidosis, type II or unspecified type, uncontrolled |
| 250.20 | Diabetes with hyperosmolarity, type II or unspecified type, not stated as uncontrolled |
| 250.22 | Diabetes with hyperosmolarity, type II or unspecified type, uncontrolled |
| 250.30 | Diabetes with other coma, type II or unspecified type, not stated as uncontrolled |
| 250.32 | Diabetes with other coma, type II or unspecified type, uncontrolled |
| 250.40 | Diabetes with renal manifestations, type II or unspecified type, not stated as uncontrolled |
| 250.42 | Diabetes with renal manifestations, type II or unspecified type, uncontrolled |
| 250.50 | Diabetes with ophthalmic manifestations, type II or unspecified type, not stated as uncontrolled |
| 250.52 | Diabetes with ophthalmic manifestations, type II or unspecified type, uncontrolled |
| 250.60 | Diabetes with neurological manifestations, type II or unspecified type, not stated as uncontrolled |
| 250.62 | Diabetes with neurological manifestations, type II or unspecified type, uncontrolled |
| 250.70 | Diabetes with peripheral circulatory disorders, type II or unspecified type, not stated as uncontrolled |
| 250.72 | Diabetes with peripheral circulatory disorders, type II or unspecified type, uncontrolled |
| 250.80 | Diabetes with other specified manifestations, type II or unspecified type, not stated as uncontrolled |
| 250.82 | Diabetes with other specified manifestations, type II or unspecified type, uncontrolled |
| 250.90 | Diabetes with unspecified complication, type II or unspecified type, not stated as uncontrolled |
| 250.92 | Diabetes with unspecified complication, type II or unspecified type, uncontrolled |
| 357.2 | Polyneuropathy in diabetes |
| E08.42 | Diabetes mellitus due to underlying condition with diabetic polyneuropathy |
| E09.42 | Drug or chemical induced diabetes mellitus with neurological complications with diabetic polyneuropathy |
| E11 | Type 2 diabetes mellitus |
| E11.0 | Type 2 diabetes mellitus with hyperosmolarity |
| E11.00 | Type 2 diabetes mellitus with hyperosmolarity without nonketotic hyperglycemic-hyperosmolar coma (NKHHC) |
| E11.01 | Type 2 diabetes mellitus with hyperosmolarity with coma |
| E11.2 | Type 2 diabetes mellitus with kidney complications |
| E11.21 | Type 2 diabetes mellitus with diabetic nephropathy |
| E11.22 | Type 2 diabetes mellitus with diabetic chronic kidney disease |
| E11.29 | Type 2 diabetes mellitus with other diabetic kidney complication |
| E11.3 | Type 2 diabetes mellitus with ophthalmic complications |
| E11.31 | Type 2 diabetes mellitus with unspecified diabetic retinopathy |
| E11.311 | Type 2 diabetes mellitus with unspecified diabetic retinopathy with macular edema |
| E11.319 | Type 2 diabetes mellitus with unspecified diabetic retinopathy without macular edema |
| E11.32 | Type 2 diabetes mellitus with mild nonproliferative diabetic retinopathy |
| E11.321 | Type 2 diabetes mellitus with mild nonproliferative diabetic retinopathy with macular edema |
| E11.329 | Type 2 diabetes mellitus with mild nonproliferative diabetic retinopathy without macular edema |
| E11.33 | Type 2 diabetes mellitus with moderate nonproliferative diabetic retinopathy |
| E11.331 | Type 2 diabetes mellitus with moderate nonproliferative diabetic retinopathy with macular edema |
| E11.339 | Type 2 diabetes mellitus with moderate nonproliferative diabetic retinopathy without macular edema |
| E11.34 | Type 2 diabetes mellitus with severe nonproliferative diabetic retinopathy |
| E11.341 | Type 2 diabetes mellitus with severe nonproliferative diabetic retinopathy with macular edema |
| E11.349 | Type 2 diabetes mellitus with severe nonproliferative diabetic retinopathy without macular edema |
| E11.35 | Type 2 diabetes mellitus with proliferative diabetic retinopathy |
| E11.351 | Type 2 diabetes mellitus with proliferative diabetic retinopathy with macular edema |
| E11.359 | Type 2 diabetes mellitus with proliferative diabetic retinopathy without macular edema |
| E11.36 | Type 2 diabetes mellitus with diabetic cataract |
| E11.39 | Type 2 diabetes mellitus with other diabetic ophthalmic complication |
| E11.4 | Type 2 diabetes mellitus with neurological complications |
| E11.40 | Type 2 diabetes mellitus with diabetic neuropathy, unspecified |
| E11.41 | Type 2 diabetes mellitus with diabetic mononeuropathy |
| E11.42 | Type 2 diabetes mellitus with diabetic polyneuropathy |
| E11.43 | Type 2 diabetes mellitus with diabetic autonomic (poly)neuropathy |
| E11.44 | Type 2 diabetes mellitus with diabetic amyotrophy |
| E11.49 | Type 2 diabetes mellitus with other diabetic neurological complication |
| E11.5 | Type 2 diabetes mellitus with circulatory complications |
| E11.51 | Type 2 diabetes mellitus with diabetic peripheral angiopathy without gangrene |
| E11.52 | Type 2 diabetes mellitus with diabetic peripheral angiopathy with gangrene |
| E11.59 | Type 2 diabetes mellitus with other circulatory complications |
| E11.6 | Type 2 diabetes mellitus with other specified complications |
| E11.61 | Type 2 diabetes mellitus with diabetic arthropathy |
| E11.610 | Type 2 diabetes mellitus with diabetic neuropathic arthropathy |
| E11.618 | Type 2 diabetes mellitus with other diabetic arthropathy |
| E11.62 | Type 2 diabetes mellitus with skin complications |
| E11.620 | Type 2 diabetes mellitus with diabetic dermatitis |
| E11.621 | Type 2 diabetes mellitus with foot ulcer |
| E11.622 | Type 2 diabetes mellitus with other skin ulcer |
| E11.628 | Type 2 diabetes mellitus with other skin complications |
| E11.63 | Type 2 diabetes mellitus with oral complications |
| E11.630 | Type 2 diabetes mellitus with periodontal disease |
| E11.638 | Type 2 diabetes mellitus with other oral complications |
| E11.64 | Type 2 diabetes mellitus with hypoglycemia |
| E11.641 | Type 2 diabetes mellitus with hypoglycemia with coma |
| E11.649 | Type 2 diabetes mellitus with hypoglycemia without coma |
| E11.65 | Type 2 diabetes mellitus with hyperglycemia |
| E11.69 | Type 2 diabetes mellitus with other specified complication |
| E11.8 | Type 2 diabetes mellitus with unspecified complications |
| E11.9 | Type 2 diabetes mellitus without complications |
| E13.42 | Other specified diabetes mellitus with diabetic polyneuropathy |
| Chronic kidney disease | |
| 403 | Hypertensive chronic kidney disease |
| 403.0 | Hypertensive chronic kidney disease, malignant |
| 403.00 | Hypertensive chronic kidney disease, malignant, with chronic kidney disease stage I through stage IV, or unspecified |
| 403.01 | Hypertensive chronic kidney disease, malignant, with chronic kidney disease stage V or end stage renal disease |
| 403.1 | Hypertensive chronic kidney disease, benign |
| 403.10 | Hypertensive chronic kidney disease, benign, with chronic kidney disease stage I through stage IV, or unspecified |
| 403.11 | Hypertensive chronic kidney disease, benign, with chronic kidney disease stage V or end stage renal disease |
| 403.9 | Hypertensive chronic kidney disease, unspecified |
| 403.90 | Hypertensive chronic kidney disease, unspecified, with chronic kidney disease stage I through stage IV, or unspecified |
| 403.91 | Hypertensive chronic kidney disease, unspecified, with chronic kidney disease stage V or end stage renal disease |
| 404 | Hypertensive heart and chronic kidney disease |
| 404.0 | Hypertensive heart and chronic kidney disease, malignant |
| 404.00 | Hypertensive heart and chronic kidney disease, malignant, without heart failure and with chronic kidney disease stage I through stage IV, or unspecified |
| 404.01 | Hypertensive heart and chronic kidney disease, malignant, with heart failure and with chronic kidney disease stage I through stage IV, or unspecified |
| 404.02 | Hypertensive heart and chronic kidney disease, malignant, without heart failure and with chronic kidney disease stage V or end stage renal disease |
| 404.03 | Hypertensive heart and chronic kidney disease, malignant, with heart failure and with chronic kidney disease stage V or end stage renal disease |
| 404.1 | Hypertensive heart and chronic kidney disease, benign |
| 404.10 | Hypertensive heart and chronic kidney disease, benign, without heart failure and with chronic kidney disease stage I through stage IV, or unspecified |
| 404.11 | Hypertensive heart and chronic kidney disease, benign, with heart failure and with chronic kidney disease stage I through stage IV, or unspecified |
| 404.12 | Hypertensive heart and chronic kidney disease, benign, without heart failure and with chronic kidney disease stage V or end stage renal disease |
| 404.13 | Hypertensive heart and chronic kidney disease, benign, with heart failure and chronic kidney disease stage V or end stage renal disease |
| 404.9 | Hypertensive heart and chronic kidney disease, unspecified |
| 404.90 | Hypertensive heart and chronic kidney disease, unspecified, without heart failure and with chronic kidney disease stage I through stage IV, or unspecified |
| 404.91 | Hypertensive heart and chronic kidney disease, unspecified, with heart failure and with chronic kidney disease stage I through stage IV, or unspecified |
| 404.92 | Hypertensive heart and chronic kidney disease, unspecified, without heart failure and with chronic kidney disease stage V or end stage renal disease |
| 404.93 | Hypertensive heart and chronic kidney disease, unspecified, with heart failure and chronic kidney disease stage V or end stage renal disease |
| 585 | Chronic kidney disease (CKD) |
| 585.1 | Chronic kidney disease, stage I |
| 585.2 | Chronic kidney disease, stage II (mild) |
| 585.3 | Chronic kidney disease, stage III (moderate) |
| 585.4 | Chronic kidney disease, stage IV (severe) |
| 585.5 | Chronic kidney disease, stage V |
| 585.6 | End stage renal disease |
| 585.9 | Chronic kidney disease, unspecified |
| 586 | Renal failure, unspecified |
| I12 | Hypertensive chronic kidney disease |
| I12.0 | Hypertensive chronic kidney disease with stage 5 chronic kidney disease or end stage renal disease |
| I12.9 | Hypertensive chronic kidney disease with stage 1 through stage 4 chronic kidney disease, or unspecified chronic kidney disease |
| I13 | Hypertensive heart and chronic kidney disease |
| I13.0 | Hypertensive heart and chronic kidney disease with heart failure and stage 1 through stage 4 chronic kidney disease, or unspecified chronic kidney disease |
| I13.1 | Hypertensive heart and chronic kidney disease without heart failure |
| I13.10 | Hypertensive heart and chronic kidney disease without heart failure, with stage 1 through stage 4 chronic kidney disease, or unspecified chronic kidney disease |
| I13.11 | Hypertensive heart and chronic kidney disease without heart failure, with stage 5 chronic kidney disease, or end stage renal disease |
| I13.2 | Hypertensive heart and chronic kidney disease with heart failure and with stage 5 chronic kidney disease, or end stage renal disease |
| N18 | Chronic kidney disease (CKD) |
| N18.1 | Chronic kidney disease, stage 1 |
| N18.2 | Chronic kidney disease, stage 2 (mild) |
| N18.3 | Chronic kidney disease, stage 3 (moderate) |
| N18.4 | Chronic kidney disease, stage 4 (severe) |
| N18.5 | Chronic kidney disease, stage 5 |
| N18.6 | End stage renal disease |
| N18.9 | Chronic kidney disease, unspecified |
| N19 | Unspecified kidney failure |

*CKD* chronic kidney disease, *ICD* International Classification of Diseases, *T2D* type 2 diabetes

**Table S2** Baseline demographic and clinical characteristics of the cohort of all patients receiving spironolactone, overall and stratified by spironolactone treatment persistence

| Characteristic | All spironolactone users (*n* = 5430) | Persistent spironolactone users  (*n* = 2800) | Non-persistent spironolactone users (*n* = 2630) |
| --- | --- | --- | --- |
| Age at inclusion (years) | | | |
| Median (range) | 61 (20–82) | 61 (20–82) | 62 (22–82) |
| Sex (%) | | | |
| Male | 60.2 | 62.1 | 58.2 |
| Ethnicity^a^ (%) | | | |
| Caucasian | 74.7 | 77.1 | 71.9 |
| African American | 14.7 | 13.9 | 15.6 |
| Hispanic | 7.0 | 5.5 | 8.7 |
| Other | 1.6 | 1.6 | 1.7 |
| Unspecified | 2.0 | 1.8 | 2.2 |
| CKD stage at inclusion (%) | | | |
| Stage 1 | 5.4 | 5.9 | 4.9 |
| Stage 2 | 10.8 | 12.5 | 8.9 |
| Stage 3 | 36.0 | 36.8 | 35.2 |
| Stage 4 | 7.0 | 5.7 | 8.3 |
| Stage 5 | 0.8 | 0.6 | 1.0 |
| ESRD/RRT | 12.8 | 10.5 | 15.2 |
| Missing | 27.3 | 28.0 | 26.5 |
| Comorbidities (%) | | | |
| Heart failure | 52.8 | 49.8 | 55.9 |
| Hypertension | 98.4 | 98.3 | 98.5 |
| CV disease | 70.3 | 68.9 | 71.9 |
| Oedema | 49.5 | 46.6 | 52.7 |
| Proteinuria | 26.5 | 27.1 | 25.9 |
| Hyperkalaemia | 12.0 | 9.7 | 14.5 |
| Annual pre-inclusion median healthcare costs (US$) | | | |
| Total costs | 56 119 | 52 071 | 60 640 |
| Inpatient costs | 17 900 | 14 382 | 21 299 |
| Outpatient costs | 17 132 | 16 490 | 18 076 |
| Pharmacy costs | 10 506 | 11 039 | 9944 |

^a^Among the subset of patients linkable to the Experian database (all spironolactone users, *n* = 1304; persistent users, *n* = 703; non-persistent users, *n* = 601)

*CKD* chronic kidney disease, *CV* cardiovascular, *ESRD* end-stage renal disease, *RRT* renal replacement therapy

**Fig. S1** **Determination of inclusion date for study cohorts.** (A) Matched users and non-users of spironolactone with DKD. (B) Cohort of spironolactone users for exploratory analysis of treatment persistence. CKD, chronic kidney disease; DKD, diabetic kidney disease; T2D, type 2 diabetes


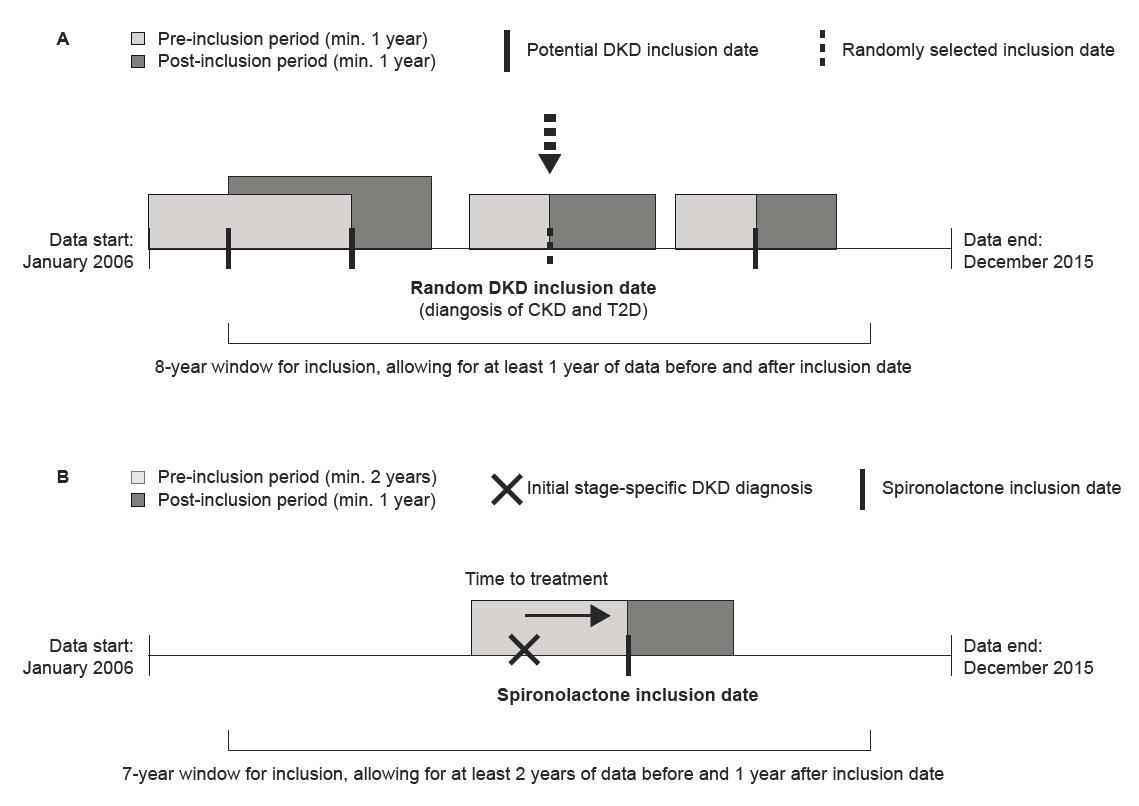


**Fig. S2 Other clinical events of interest occurring in the post-inclusion period in the matched cohorts of spironolactone users and non-users.** A 60-day gap was used to count acute events (stroke [ischaemic], revascularization, and hyponatraemia), a 360-day gap was used to count chronic events (proteinuria and reproductive system and breast disorders), and a 1-day gap was used for other events (amputation)


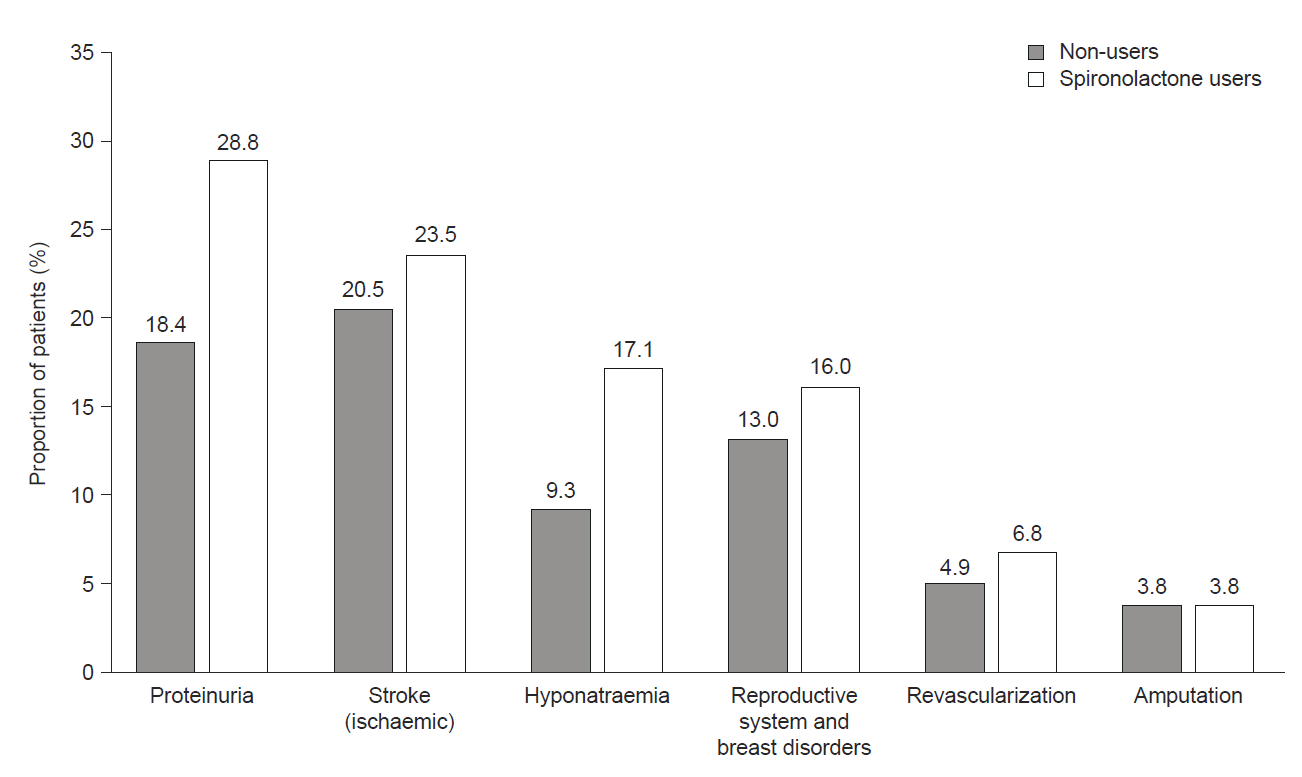


**Fig. S3 Progression to a more advanced stage of CKD, ESRD, or RRT in 1-year post-inclusion in persistent and non-persistent users of spironolactone.** CKD, chronic kidney disease; ESRD, end-stage renal disease; RRT, renal replacement therapy


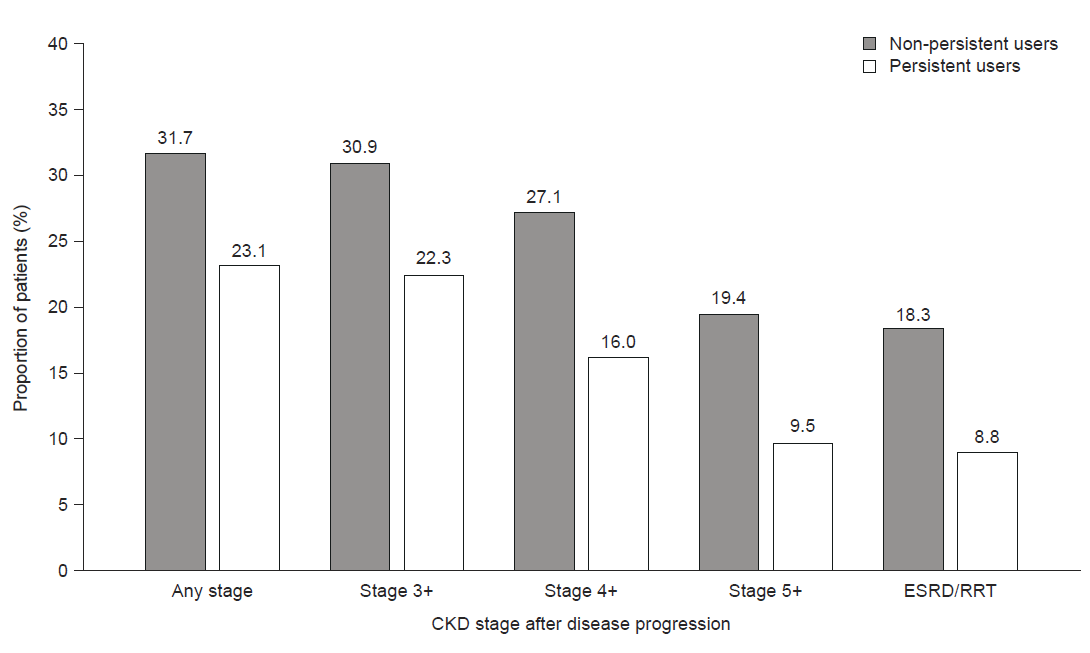


**Fig. S4 Clinical events of interest occurring in the post-inclusion period in persistent and non-persistent users of spironolactone.** A 60-day gap was used to count acute events [ACS, acute kidney injury, stroke (any), HF and hyperkalaemia] and a 360-day gap was used to count chronic events (PAD and diabetic retinopathy). ACS, acute coronary syndrome; AKI, acute kidney injury; HF, heart failure; PAD, peripheral artery disease


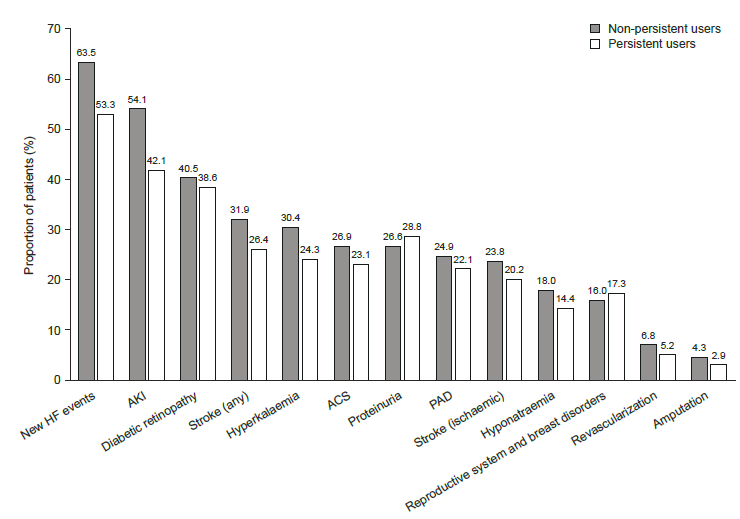

Supplement: Supplementary file 1 — Additional file 1 Table S1. List of codes used to identify eligible patients with CKD and T2D. Table S2. Baseline demographic and clinical characteristics of the cohort of all patients receiving spironolactone, overall and stratified by spironolactone treatment persistence. Figure S1. Determination of inclusion date for study cohorts. (A) Matched users and non-users of spironolactone with DKD. (B) Cohort of spironolactone users for exploratory analysis of treatment persistence. CKD, chronic kidney disease; DKD, diabetic kidney disease; T2D, type 2 diabetes. Figure S2. Other clinical events of interest occurring in the post-inclusion period in the matched cohorts of spironolactone users and non-users. A 60-day gap was used to count acute events (stroke [ischaemic], revascularization, and hyponatraemia), a 360-day gap was used to count chronic events (proteinuria and reproductive system and breast disorders), and a 1-day gap was used for other events (amputation). Figure S3. Progression to a more advanced stage of CKD, ESRD, or RRT in 1-year post-inclusion in persistent and non-persistent users of spironolactone. CKD, chronic kidney disease; ESRD, end-stage renal disease; RRT, renal replacement therapy. Figure S4. Clinical events of interest occurring in the post-inclusion period in persistent and non-persistent users of spironolactone. A 60-day gap was used to count acute events [ACS, acute kidney injury, stroke (any), HF and hyperkalaemia] and a 360-day gap was used to count chronic events (PAD and diabetic retinopathy). ACS, acute coronary syndrome; AKI, acute kidney injury; HF, heart failure; PAD, peripheral artery disease. [file 12882_2020_1719_MOESM1_ESM.docx]
